# Professional mathematicians differ from controls in their spatial-numerical associations

**DOI:** 10.1007/s00426-015-0677-6

**Published:** 2015-06-11

**Authors:** Krzysztof Cipora, Mateusz Hohol, Hans-Christoph Nuerk, Klaus Willmes, Bartosz Brożek, Bartłomiej Kucharzyk, Edward Nęcka

**Affiliations:** Institute of Psychology, Jagiellonian University, Ul. Ingardena 6, 30-060 Cracow, Poland; Copernicus Center for Interdisciplinary Studies, Cracow, Poland; Department of Philosophy, Pontifical University of John Paul II, Cracow, Poland; Department of Psychology and LEAD Graduate School, University of Tuebingen, Tübingen, Germany; Knowledge Media Research Center, IWM-KMRC, Tübingen, Germany; Section Neuropsychology, Neurological Clinic, RWTH Aachen University, Aachen, Germany; Department of Philosophy of Law and Legal Ethics, Jagiellonian University, Cracow, Poland

## Abstract

While mathematically impaired individuals have been shown to have deficits in all kinds of basic numerical representations, among them spatial-numerical associations, little is known about individuals with exceptionally high math expertise. They might have a more abstract magnitude representation or more flexible spatial associations, so that no automatic left/small and right/large spatial-numerical association is elicited. To pursue this question, we examined the Spatial Numerical Association of Response Codes (SNARC) effect in professional mathematicians which was compared to two control groups: Professionals who use advanced math in their work but are not mathematicians (mostly engineers), and matched controls. Contrarily to both control groups, Mathematicians did not reveal a SNARC effect. The group differences could not be accounted for by differences in mean response speed, response variance or intelligence or a general tendency not to show spatial-numerical associations. We propose that professional mathematicians possess more abstract and/or spatially very flexible numerical representations and therefore do not exhibit or do have a largely reduced default left-to-right spatial-numerical orientation as indexed by the SNARC effect, but we also discuss other possible accounts. We argue that this comparison with professional mathematicians also tells us about the nature of spatial-numerical associations in persons with much less mathematical expertise or knowledge.

## Introduction

### The SNARC effect and possible underlying mechanisms

Dehaene, Bossini, and Giraux (1993) found that in a speeded bimanual decision task, participants responded faster to small magnitude numbers with their left hand and to large magnitude numbers with their right hand. The acronym SNARC, Spatial-Numerical Association of Response Codes, is used to label this phenomenon. Later studies showed that this leftward bias towards small magnitude numbers and rightward bias towards large magnitude numbers are not limited to bimanual responses and extend to unimanual tasks, as well as to responding with feet or eye movements (see Wood, Willmes, Nuerk, & Fischer, 2008 for a review and meta-analysis; Gevers & Lammertyn, 2005; see also Fischer & Shaki, 2014 for a current overview).

The SNARC effect has been interpreted as providing evidence that number magnitude representations are mapped spatially. Its presence is usually considered to be a signature of automatic spatial processing of numerical magnitude. However, despite 20 years of extensive research on the SNARC effect, there is no consensus about the underlying mechanisms. Most popular explanations refer to (1) cultural factors, in particular reading and counting direction (see e.g., Zebian, 2005; Shaki, Fischer, & Petrusic, 2009), (2) factors related to the spatial mapping of numbers limited to the ongoing task (Bächtold, Baumüller, & Brugger, 1998; Fischer, Mills, & Shaki, 2010), (3) finger counting habits per se (Fischer, 2008), (4) ordering of elements in working memory during a task (van Dijck & Fias, 2011), (5) dichotomous verbal coding of polar adjectives and mapping this code to spatial locations (Gevers et al., 2010; Nuerk, Iversen, & Willmes, 2004; Proctor & Cho, 2006), and (6) attentional factors (Nuerk, Bauer, Krummenacher, Heller, & Willmes, 2005b). Some of these factors (1–3) have been integrated in a recently proposed theoretical framework about the embodiment of numerical representations, showing how grounding, embodiment, and situatedness influence the spatial representations of numbers (Fischer & Brugger, 2011; Fischer, 2012; see also Wasner, Moeller, Fischer, & Nuerk, 2014). While it is still not clear which of the above mechanisms contribute to the SNARC effect, the observation that numbers are somehow associated with spatial response mappings has been replicated numerous times.

### Interindividual differences in SNARC effect

Group and interindividual differences were also observed for the SNARC effect with regard to some of the factors just mentioned: For instance, in Western cultures, only 70 % participants reveal a left-to-right SNARC effect, but 30 % descriptively show the reverse (see Wood et al., 2008; Cipora & Nuerk, 2013; Hoffmann, Pigat, & Schiltz, 2014a; Hoffmann, Mussolin, Martin, & Schiltz, 2014b). SNARC also varies with individual reading habits, both in general and in the experimental situation (Fischer, Shaki, & Cruise, 2009). Moreover, individual finger counting habits (Fischer, 2008; Lindemann, Alipour, & Fischer, 2011) and some inhibition capacities influence the SNARC effect (Hoffmann et al. 2014b). Other factors are age (meta-analysis of Wood et al., 2008; Hoffmann et al., 2014b) and sex (Bull, Cleland, & Mitchell, 2013). However, the difference is very small and its detection requires relatively large groups (20 participants per group).

Recent evidence shows that the magnitude of the SNARC effect is also related to mental rotation effects as well as to numerical distance effects (Viarouge, Hubbard, & McCandliss, 2014). The same study shows that participants revealing a strong mental rotation effect (i.e., larger increase in RT with the increasing rotation angle) as well as those who reveal a large distance effect (i.e., a larger increase in RT when comparing numbers that are numerically close than when comparing numbers that are numerically distant) reveal a stronger SNARC effect. In other words, participants whose spatial abilities are poorer and who have a less precise number magnitude representation seem to associate number magnitude with space more strongly. Finally, the SNARC effect has been linked to arithmetic skills, but the findings are inconsistent (Dehaene et al., 1993; Fischer & Rottmann, 2005; Cipora & Nuerk, 2013; Hoffmann et al., 2014a) and will be discussed below in more detail.

Beyond such individual and group differences, individual response characteristics associated with the experiment itself constitute the most robust factors influencing the SNARC effect (Cipora & Nuerk, 2013; Gevers, Verguts, Reynvoet, Caessens, & Fias, 2006; Wood et al., 2008, for a meta-analysis). Longer responses and higher intraindividual variability in a parity judgement task are associated with larger SNARC effects.

Nevertheless, although the SNARC effect was first studied as a general cognitive effect, reflecting automatic activation of spatial-numerical associations, individual differences are ubiquitous and sometimes show that general statements about spatial-numerical associations (particularly, the spatial mapping of number representations and its directionality) must be further differentiated or even occasionally reversed.

### Is number magnitude representation always associated with space?

Numerical representations can operate without a spatial component. As shown by Helmreich et al. (2011) or Nunez, Doan, and Nikoulina (2011), the spatial component of magnitude representation is not obligatory. There is evidence for an innate magnitude representation (showing properties such as logarithmic compression), but its spatial mapping seems to be mediated by cultural factors. Numerical magnitude can in principle be processed semantically without evoking a spatial-directional component (Nuerk et al., 2005a, for data; see Patro, Nuerk, Cress, & Haman, 2014, for a model taxonomy and data from children).

### Math expertise and elementary number processing: different research perspectives

Because we are reporting on a study of (directional) spatial-numerical associations in professional mathematicians, we will give a short overview of what is known about their specific characteristics as compared to controls. Studies are rare, but overall they seem to imply that similarities and differences between highly math-skilled participants and the general population refer both to the general personality and cognitive characteristics as well as to domain-specific factors like elementary number processing.

Professional mathematical expertise refers to problem-solving and logical-deductive thinking in highly abstract spaces and structures (i.e., finding mathematical proofs or counterexamples to theorems), but not necessarily to arithmetic skills and calculation. In fact, Pesenti (2005) showed systematically that successful mathematicians do not have to be exceptional calculators.^1^ Skills of exceptional calculators are very often limited to arithmetic and they are not capable of conducting mathematical proofs.

The first preliminary evidence for some unique abilities in highly math-skilled individuals can be derived from research and theoretical reasoning about cognitive development, especially from Piagetian theory. Kuhn, Langer, Kohlberg, and Haan (1977) observed that only 30 % of adults reach the formal operation stage, which comprises the ability to think about abstract concepts and to use formal logic principles for reasoning, irrespective of the content of problems to be solved. By definition, this ability is fundamental and necessary for professional mathematics; therefore, all professional mathematicians should have mastered formal operations. This already suggests that as a population they are slightly different from the general population. Fluid intelligence studies also corroborate this assumption: Professional mathematicians are characterized by above average intelligence; 50 % of the variance in math achievement can be accounted for by fluid intelligence (see e.g., Wei, Yuan, Chen, & Zhou, 2012). Complex problem solving (CPS) skills (Sonnleitner, Keller, Martin, & Brunner, 2013) usually measured with computerized Microworld tasks may also be very relevant in mathematical expertise. This set of abilities, such as rule identification, rule knowledge, and rule application, can be predictive of academic success and go beyond factors that are assessed by traditional intelligence measures. These higher order thinking skills correlate moderately with intelligence as well as with math achievement and may play a particular role in tasks that are performed by professional mathematicians.

Arithmetic (but not necessarily mathematical, see above) expertise was also a subject of direct investigation, and studies conducted up to date can be classified into several groups. First of all calculation prodigies were examined (e.g., Fehr, Weber, Willmes, & Herrmann, 2010; Fehr, Wallace, Erhard, & Herrmann, 2011; Pesenti, 2005 for comparison). The studies suggest that extraordinary calculation skills mostly require drill and employ the same neural circuits as in normal participants. On the other hand, calculation prodigies are characterized by their excellent working memory capacity (usually being restricted to the numerical domain; Fehr et al., 2010; Pesenti, 2005). In several cases, calculation prodigies were diagnosed with autism or Asperger syndrome. Non-savant prodigies also differ from the general population in several personality traits (e.g., a calculation prodigy studied by Fehr et al., 2010 had higher neuroticism, aggressiveness, conscientiousness, and openness to experience than matched controls). On the other hand, his general IQ was in the normal up to moderately above normal range. In line with other studies mentioned above, this seems to suggest once more that high arithmetic skills are not necessarily linked to high mathematical expertise.

More recent lines of research addressed relations between mathematical/arithmetic expertise and elementary numerical processing, but led to inconsistent results. The most prominent scheme is to compare students of math-related fields of study with students of humanities or social sciences with respect to elementary numerical processing. Such an attempt was made by Castronovo and Göbel (2012), who showed that math-skilled participants do not differ from their less-skilled peers in comparing non-symbolic numerosities (sets of dots). However, skilled participants outperformed controls in matching numbers to non-symbolic numerosities as well as in comparing numbers. Wei et al. (2012) observed no relationship between elementary number processing (comparison of dots of two arrays; estimation of numerosity; number comparison; etc.) and the ability to acquire advanced math concepts, but measures of elementary numerical processing were correlated with elementary math performance. Hanson and Hogan (2000) show that estimation skills at the college age correlate with the SAT mathematics score but not with the SAT verbal score.

To summarize, the results are very inconsistent. Apart from task differences, one reason may be that the above-mentioned studies show differences in elementary number processing depending on the arithmetic skill level observed. Participants were students from different faculties. These inclusion criteria do not guarantee a high level of math expertise, because even in many science departments students are often more concerned with calculation and application of formulae than with mathematical proofs.

Highly notable exceptions are two studies by Dowker (1992) and Dowker, Flood, Griffiths, Harriss, and Hook (1996), who examined professional mathematicians ranging from postdocs to successful professors. Mathematicians were asked to estimate the result of multi-digit multiplications (which in fact is not considered a typical measure of elementary numerical processing). In this arithmetic task, they outperformed control groups consisting of experienced accountants and psychologists. The reason seemed to be that the mathematicians used a much wider range of strategies for estimation. Professional mathematicians were also more flexible in their strategy use (when faced with the same problem after a few months delay, they used novel strategies) and were less afraid of making mistakes than controls. They reported feelings of joy when playing with numbers. The authors concluded that a mathematician’s work is based mostly on experimentation when facing novel problems they have never solved before (and without information of whether a given problem has a solution).

Another line of evidence comes from introspective reports of professional mathematicians. Penrose (1989) claims that solving mathematical problems is not based solely on using algorithms. The process of deciding which algorithm should be applied plays a crucial role in most cases.

Penrose also introspectively reports that a considerable part of mathematical thinking takes the form of operations on mental entities, which are sometimes hard to verbalize. On the other hand, he reports that his colleagues think very differently compared to him; so, there may be very high interindividual variability in thinking in the group of professional mathematicians.

To sum up, the demands of elaborating new ways of facing and solving novel mathematical problems may enhance the development of more flexible numerical representations in professional mathematicians. It has not been studied whether mathematicians are also different in basic spatial-numerical associations.

### The relation between arithmetic/mathematical skills and SNARC

Because there are no data about professional mathematicians, we will review the literature about the relation of arithmetic/mathematical skills and the SNARC to derive our hypothesis. Originally, participants who are more advanced in math were thought to reveal a smaller SNARC effect than those who were less proficient. The first evidence came from Experiment 1 in the seminal study by Dehaene et al. (1993). There was a non-significant tendency that math-skilled participants (*n* = 10) revealed a smaller SNARC effect than their non-skilled peers (*n* = 10). The level of math skills was operationalized by what field of study (science vs humanities, mostly literature; for a more thorough discussion of results of this experiment refer to Cipora & Nuerk, 2013) was attended by the participants. Despite its lack of significance, this result was cited as an indication that math-skilled participants reveal a smaller SNARC effect (e.g., Fischer et al., 2010; Fischer & Rottmann, 2005). Fischer and Rottmann (2005) again raised the issue of the relation between math skill and the SNARC effect. Comparing two groups (10 participants in each, grouping based on field of study: mathematics, physics, engineering vs psychology), they found a numerical tendency towards a difference, which again was far from statistical significance (*p* = .28). Moreover, in a study on the mental representation of fractions, Bonato, Fabbri, Umiltà, and Zorzi, (2007) in Experiment 1 also compared math-skilled and non-skilled participants (groups of 10, students of engineering and psychology). In this study, there was no difference between skilled and non-skilled participants in the magnitude of the SNARC effect. A similar result was reported in Experiment 4 of that study.

So, while there were some tendencies towards smaller SNARC effect in math-skilled participants, they all remained non-significant. However, all of the studies mentioned above have some limitations affecting their conclusiveness. First of all, the groups were very small, which raises serious concerns about inferential statistical power. Cipora and Wood (2012) showed that detecting between-group differences in SNARC seems to be very difficult and issues of statistical power have to be addressed properly (increasing the number of repetitions of a single stimulus, relatively large groups). Secondly, apart from the study by Dehaene et al. (1993), the studies cited above used modified versions of the typical parity judgment task. Fischer and Rottmann (2005) included negative numbers in their experiment (regarding the influence of the particular number stimuli set on the SNARC effect see Dehaene et al., 1993, Experiment 3, van Dijck & Fias, 2011). Bonato et al. (2007) in their study used fractions that are processed in a slightly different way than integers (Schneider & Siegler, 2010). Therefore, a lack of between-group differences can possibly be accounted for by the unique nature of the material used in a given study. Third, group inclusion criteria were based on the field of study chosen. In case of small groups, it is possible that there were several skilled participants in the presumably non-skilled groups. There was no external measure of math skill to control skill level. Fourth, there was no theoretical background on what kind of skill may be related to the SNARC effect (abstract representations, calculation fluency, etc., see Cipora & Nuerk, 2013 for discussion).

Fortunately, some recent studies have addressed one or several of these points. Schneider, Grabner, and Paetsch (2009) examined relations between basic signatures of numerical processing and school achievement in 5th and 6th grades. Besides other findings, they observed no relationship between the SNARC effect and math achievement in a large sample of more than 400 participants: There were no significant correlations between the SNARC effect and math grades, number line estimation accuracy, total score for the numerical subscale in a standardized cognitive abilities test (KFT), nor conceptual knowledge about math. Moreover, there was an external criterion for math skill (school achievement). However, this work was conducted with children and because SNARC effects develop with age (Berch, Foley, Hill, & Ryan, 1999; Wood et al., 2008), we do not know whether these results will hold for adults.

Bull et al. (2013) conducted a study on gender differences in the SNARC effect. In Experiment 2, they tested 40 participants, twice as many as previous studies. The participants performed a modified color discrimination task, which was used to measure the SNARC effect. Math competence was assessed by the Numerical Operations subtest from the Wechsler Wide Achievement Test (WIAT II, UK). In this study, virtually no relation between SNARC and math ability was found (*r* = −.07). However, this study might not be representative for SNARC effects, because the authors used a non-semantic task, i.e., no judgement about semantic attributes of numbers (e.g., parity) was required.

Very recently, two studies were published with adults and featuring typical SNARC tasks and large samples, thereby reducing power issues. Cipora and Nuerk (2013) examined 71 participants comparing students studying math-related subjects (math, computer studies, engineering, etc.; *n* = 18) and those studying subjects in which math is not crucial (psychology, literature etc.; *n* = 53). The mean age of participants was below 22 years old; therefore, they were mostly at the beginning of their studies. Despite the relatively large sample sizes and extensive efforts to control power and reliability issues, there was no relationship between arithmetic skill nor field of study with the magnitude of the SNARC effect. Nevertheless, their sample was not gender balanced and proportions of males and females differed between skill groups. Furthermore, the presumably skilled group comprised far fewer participants than the presumably non-skilled group. Hoffmann et al. (2014a) also tested a large group (*n* = 95) comprised 3 groups of university students: (1) “Math expert” refers to students “with a study field having a strong numerical load (e.g., mathematics, engineering, and sciences),” (2) controls, and (3) a math difficulties group. Again, participants were students (mean age 23.2 years) and no professional mathematicians. In contrast to Cipora and Nuerk (2013), a between-group difference was observed, but its effect size was small (partial *η*^2^ = 0.05). Nevertheless, considerable differences in design, especially the inclusion of zero in the stimulus set (for a specific role of zero see: Nuerk et al., 2004), do not allow for direct comparison with the study by Cipora & Nuerk (2013). However, in the Hoffmann et al. (2014a) study, the between-group difference in the SNARC effect remained, when 0 and 5 were excluded from the analyses: the highly skilled math group still differed significantly from the controls. The correlation between the SNARC slope and arithmetic even remained when the math difficulties group was excluded from their analyses. When these analyses are considered, some substantial differences between the results of both studies remain.

In sum, 6 out of 7 studies (Bonato et al., 2007; Bull et al., 2013; Cipora & Nuerk, 2013; Dehaene et al., 1993; Fischer & Rottmann, 2005; Schneider et al., 2009) observed no significant relation between SNARC and arithmetic competence (directly assessed or indirectly via field of study), while one recent study (Hoffmann et al., 2014a) observed a significant, albeit small relation. None of these SNARC studies used professional mathematicians as studies examining other questions had done (Dowker, 1992; Dowker et al., 1996).

### Objectives of the presented study

As we have argued in the previous sections, the origin of interindividual differences in the SNARC effect is largely unknown even after over 20 years of research. Throughout these years, the math skill level was one of the most interesting factors to be taken into consideration. Since it seemed to be difficult to find a relation between SNARC slopes and arithmetic skill in the typical skill range, the next step is to examine extreme groups. This is the approach we are taking in this study.

Because Hoffmann et al. (2014a) had already studied participants with math difficulties, we now decided to explore the SNARC effect at the other end of the spectrum, i.e., in professional math experts. We not only used math students (who have not finished their studies, and whose math expertise may or may not be so good), nor did we stop at examining participants with a math B.Sc. or M.Sc. degree. Rather, we used advanced Ph.D. students, who were researching mathematics as part of their daily life and who had done so for at least 3 years. In this way, we ensured a level of professional math experience, which has very rarely (see Dowker, 1992) been studied in any numerical cognition work. Examining expert mathematicians seems promising because one can expect that possible differences will be more pronounced than in case of typical skill levels for several reasons. First, professional mathematicians are required to manipulate abstract concepts in several ways in order to solve problems. Therefore, they might have a more abstract concept of numbers than typical people and might not automatically associate numbers with space. Second, mathematicians are experienced in mapping numbers and mathematical concepts to space in a very flexible and variable way. Therefore, they might exhibit spatial associations with numbers but these associations may not be consistent with a given cultural convention. Rather their space-number associations might be highly flexible, corresponding to the flexible use of space number relations in their professional work. Both accounts would predict a smaller or a null default spatial-numerical association in mathematicians.

Moreover, we decided to distinguish professional mathematicians from professionals in other fields (e.g., engineering), who use advanced arithmetic in their work but are either not interested in the study of mathematics itself or at least less so. Usually, groups employing true mathematics (working on theorems) and groups applying advanced mathematics for arithmetic are not distinguished and this will also be done here. Finally, we also used a professional control group from social sciences and humanities, who have even less experience with numbers.

In order to ensure the reliable estimation of the SNARC effect and to obtain optimal power to detect a SNARC effect, as well as individual differences in SNARC, we utilized a procedure involving many more repetitions of each number/condition (see Cipora & Nuerk, 2013; Cipora & Wood, 2012). This seems particularly important since recent evidence shows that the reliability of SNARC slopes obtained in a typical setup is rather poor (correlations of slopes <0.4; Viarouge et al., 2014; see also Wood, Nuerk, & Willmes, 2006), and hence probably reduces correlations with external measures. It is particularly noteworthy that reliability problems are not limited to the SNARC effect but are present in other chronometric measures as well (e.g., Maloney, Risko, Preston, Ansari, & Fugelsang, 2010, for the numerical distance effect). For these reasons, increasing the number of repetitions is crucial when RT difference scores are calculated (see Miller & Ulrich, 2013; for a theoretical account and some simulations). Additionally, we decided to control for fluid intelligence to ensure that any relationship between mathematical expertise and the SNARC effect was not due to related differences in fluid intelligence. When addressing the possible influences of intelligence on spatial-numerical associations, we decided to utilize a measure of fluid intelligence, namely Advanced Raven Matrices. We were especially interested in nonverbal abilities and reasoning skills for nonverbal material. Moreover, aiming at studying highly educated participants, we had to choose a measure capable of differentiating participants at highly above-average levels of fluid intelligence and avoiding ceiling effects. Among the measures of fluid intelligence available in Polish, only the Advanced Raven Matrices are suited to assess fluid intelligence at levels, which are far above average. Unfortunately, there is no Polish adaptation of the Raven Vocabulary Scales designed to complement the matrices. Time constraints also did not allow us to utilize other intelligence measures.

To ensure that we were really looking at differences regarding the association between number magnitude and space and not only at general differences of numerical effect sizes between groups, we also explored a related, yet different effect, the Linguistic Markedness Association of Response Codes (MARC) effect. The MARC effect was assumed to be driven by linguistic properties (namely linguistic markedness) of number attributes (Nuerk et al., 2004; for a current overview and theoretical frameworks for MARC effect see Huber et al., 2014b).

If a systematic relation between SNARC and professional math expertise exists, math professionals should show a smaller or a null SNARC effect (as some studies indicated descriptively smaller SNARC effects in groups partially consisting of math students). If this relation does not differ between individuals at all, no group differences should be observed. Any conclusive group difference should not be explained by control variables like intelligence and overall mean RT or RT variability, and it should be specific to the SNARC effect.

## Methods

### Participants

44 participants (6 female) took part in the study. The mean age was 27.9 years (SD = 1.1; range 26–31 years). All participants (native Polish speakers) were doctoral students (third year or higher). The inclusion criterion for our sample was to be advanced in doctoral studies, so that the exact dissertation topic has been officially approved by the department’s council. Participants constituted three groups: (1) mathematicians (*M*; *n* = 14, 2 females)—doctoral students in mathematics; (2) engineers (*E*; *n* = 15, 2 females)—doctoral students in other fields who are not professional mathematicians but use advanced math in their work (e.g., communication, chemistry, etc.); and (3) controls (*C*; *n* = 15; 2 females)—doctoral students in the humanities and social sciences (e.g., philosophy, sociology, psychology, etc.)^2^. The groups did not differ with respect to age (*M* = 28.2; *E* = 28.1; *C* = 27.5 years). All participants were right handed (due to requirements for a subsequent fMRI study—not reported here) and had normal or corrected-to-normal vision.

### Materials

A computerized parity judgment task was utilized. Participants were asked to decide on the parity of numbers presented on the screen using the P and Q keys on a standard computer keyboard. Both speed and accuracy were stressed. The task comprised two blocks with response key mapping reversed. The order of blocks was counterbalanced across participants. Numbers 1, 2, 3, 4, 6, 7, 8, and 9 were used. Black stimuli (font size 30) were presented against light gray background (210 210 210 in RGB notation) to avoid sharp contrasts. Each number was presented 30 times within each block. Each block was preceded by a training session (16 trials) to familiarize participants with the task. During the training session, accuracy feedback was provided and the required response mapping was indicated in the bottom line of the screen. The order of trials was randomized with the restriction that each number could not appear more than two times in a row.

Each trial started with an eye fixation cross presented for 300 ms. Subsequently, the number appeared and was presented until the participants responded or for a maximum duration of 2 s. The next trial started after 500 ms.

A standard, portable, MS Windows compatible computer (15.4 in.) running DMDX software (Forster & Forster, 2003) was used to present stimuli and collect responses. We also administered Advanced Raven Matrices (see Raven, Court, & Raven, 1983, Polish adaptation by Jaworowska & Szustrowa, 1991) in order to control for fluid intelligence.

### Procedure

The parity judgment task was performed as a first task before an fMRI experiment, which was not related to the SNARC effect (and is not reported here). Participants were tested individually. After informed consent had been obtained, participants were seated in front of the computer and asked to read the instructions carefully. All questions were answered if needed. The parity judgment task lasted about 12 min. After completion of the fMRI study, participants solved the Advanced Raven Matrices, results of which were used as a covariate in the present study. Series 1 was administered first in a separate booklet in order to familiarize the participants with the test. The time limit for series 1 was 5 min and the score was not analyzed further. Subsequently, participants were assessed with series 2. The time limit was 20 min.

### Data preparation

Data from training series was not analyzed. The average error rate was 3.1 %, but errors were not analyzed further. Reaction times (RTs) shorter than 200 ms (less than 0.01 % of trials) were treated as anticipations and discarded from further analyses. To solve the problem of outlier RTs, a sequential filtering procedure was applied. Mean RTs, as well as standard deviations, were calculated for each participant separately. Subsequently, RTs outside ±3 SD from a participant’s mean were discarded. Means and SDs were calculated again and this procedure was repeated until there were no more changes in mean and SD. 91.9 % of the data were retained after filtering and were then analyzed further (see also Cipora & Nuerk, 2013, for the same trimming method).

### SNARC effect calculation

In order to calculate the SNARC effect, the method proposed by Fias, Brysbaert, Geypens, and d’Ydewalle (1996) was used. It enables the calculation of the magnitude of the SNARC for each participant and produces a single numerical value, which is suitable for further comparisons. First, dRT (RT right hand − RT left hand) is calculated for each number for each participant separately. Positive dRT values indicate left-hand advantage, whereas negative dRT values indicate right-hand advantage. Subsequently, dRT values are regressed on number magnitude. Non-standardized regression slopes are taken as a measure of the SNARC effect. A more negative slope corresponds to a stronger SNARC effect. To examine whether there is a significant SNARC effect at the sample level, slopes are tested against zero with the one-sample *t* test. Since there is direct prediction regarding directionality of the SNARC effect, one-sided test for negative values can be used. This method is the most popular in the literature, so that we decided to use it as a primary measure. Additionally, we calculated multiple regressions within each participant with two predictors—number parity and magnitude—such that apart from the SNARC effect we were capable of estimating the MARC effect (right-hand advantage for even numbers and left-hand advantage for odd numbers; Nuerk et al., 2004; Nuerk, Wood, & Willmes, 2005a). This also allowed us to examine the individual fit of the regression model (*R*^2^) and the size of residuals.

Using the non-standardized regression slope as a measure of the SNARC effect size has been strongly criticized recently (Pinhas, Tzelgov, & Ganor-Stern, 2012). The non-standardized slope does not carry information regarding the fit of the regression slope to the data. According to this view, it cannot serve as a measure of SNARC effect size. Hoffmann et al. (2014a) proposed an alternative solution by calculating the within-participant Pearson correlation between dRT and number magnitude as a univariate measure of the SNARC effect. This method was also applied here. These correlation coefficients were then Fisher-Z transformed to bring their distribution closer to a normal distribution for further statistical comparisons (this measure is further referred to as standardized slope). Note that these standardized slopes (before Fisher-Z transformation) are numerically equivalent to standardized regression slopes from the univariate regression analysis used for calculating non-standardized intraindividual slopes.

## Results

### Measure reliability

In a first step, we estimated the reliability of the measures we included in our analyses. Reliable measurement is a prerequisite for the meaningful interpretation of our correlation analyses of chronometric measures (cf. Maloney et al., 2010; Miller & Ulrich, 2013). In case of mean RT, SD of RT, SNARC slopes, and standardized slopes, the split-half method (odd–even) was applied. Subsequently, we applied the Spearman-Brown adjustment to obtain the reliability estimate for the whole set of items. Regarding Advanced Raven Matrices, we also decided to estimate reliability, using Cronbach’s alpha, since the psychometric evaluation of the Polish adaptation did not comprise such specific groups as our mathematicians and engineering groups. All reliability estimates were highly satisfactory and allowed for a subsequent interpretability of correlation coefficients (see Table 1).

**Table 1:** Reliability estimates of fluid intelligence, RT characteristics, and SNARC measures

Measure	Reliability estimate	Method of estimation
Raven score	0.833	Cronbach alpha
Mean RT	0.996	Split-half, Spearman-Brown
SD (RT)	0.985	Split-half, Spearman-Brown
Non-standardized SNARC slope	0.820	Split-half, Spearman-Brown
Standardized SNARC slope	0.742	Split-half, Spearman-Brown

### Reaction time characteristics

Mean reaction time was 533 ms. Mean intraindividual SD of RT was 97 ms. Cipora and Nuerk (2013) showed that intraindividual variability in response latencies correlates with the magnitude of the SNARC effect. Neither mean RT nor intraindividual RT variability differed between groups (both *F* values <1). For RT, the group means were mathematicians (*M*) = 515 ms, engineers (*E*) = 550 ms, controls (*C*) = 532 ms, whereas for intraindividual variability the means were *M* = 94 ms, *E* = 97 ms, *C* = 101 ms.

### Fluid intelligence

For one participant, the score for the Advanced Raven Matrices was not available (because of technical problems during fMRI scanning preceding administration of the Raven test). Mean score in the Advanced Raven matrices was 27.5 (SD = 4.88). Scores for the three groups were *M* = 30.93 (SD = 4.75), *E* = 26.57 (SD = 4.27), *C* = 25.27 (SD = 3.94). The between-group mean difference was significant *F*_2,40_ = 6.73, MSE = 18.68, *p* = .003, partial *η*^2^ = 0.25. Follow-up *t* tests indicated higher fluid intelligence in professional mathematicians compared to the other two groups taken together (mean = 25.90, SD = 4.08) *t*_41_ = 3.59; *p* = .001. More conservative post hoc comparisons (with Bonferroni-Holm correction) revealed significant differences between the *M* and the *C* group (*p* = .003) as well as the *M* and the *E* group (*p* = .022). The difference between the *E* and the *C* group was not significant (*p* = .421). All these results indicate higher fluid intelligence in professional mathematicians compared to the other groups.

### SNARC slopes

As expected, we found a significant SNARC effect at the whole sample level. Mean slope was −5.06 (SD = 7.20; ranging from −25.58 to 12.92), and it differed significantly from 0 (*t*_43_ = −4.66, *p* < .001; one-sided)^3^. A total of 35 out of the 44 participants revealed negative SNARC slopes.

In the next step, we compared slopes across groups using one-factor ANOVA. Groups differed significantly in the magnitude of the SNARC effect (*F*_2,41_ = 3.65, MSE = 46.19, *p* = .035, partial *η*^2^ = 0.15). To examine whether the SNARC effect varied as a function of math expertise, we carried out the nonparametric Jonckheere-Terpstra (JT) test, which examines whether group medians increase monotonically. The JT test was chosen because we had a specific expectation regarding an ordered sequence of group SNARC slope medians decreasing with math expertise. We did not use polynomial contrasts (e.g., linear), because they assume equidistant differences between groups and we cannot be sure about that, but only about monotonic ordinal differences in skill and experience. The JT test revealed a significant decrease in slope with the increasing math expertise (*p* = .018). Note that less negative slopes refer to smaller SNARC/no SNARC. Additionally, we performed post hoc pairwise comparisons (with Bonferroni-Holm correction), which indicated that the *M* group differed significantly from the *C* group (*p* = .030). The *E* group did not differ from the *M* group (*p* = .210) or from the *C* group (*p* = .300).

In order to investigate whether non-significant differences between the *E* and *C* as well as the *M* and *E* groups were due to lack of statistical power, a Bayesian analysis as recommended by Masson (2011) was performed (see Cipora & Nuerk, 2013, for a similar application as regards the SNARC effect). This analysis provides information whether the data support the null hypothesis model (no between-group difference) or the alternative hypothesis model (between-group difference). Separate univariate ANOVAs were conducted in order to compare the *E* with the *M* as well as the *C* with the *E* group. Sums of squares were used to calculate posterior probabilities. In case of comparing the *C* with the *E* group, the analysis shows 0.67 probability in favor of the null hypothesis model (and a complimentary 0.33 probability in favor of the alternative hypothesis). The comparison of the *E* group with the *M* group revealed 0.65 probability in favor of the null hypothesis model (and a complimentary 0.35 probability in favor of the alternative hypothesis). According to guidelines proposed by Masson (2011), these results can be interpreted as weak evidence in favor of the null hypothesis in both cases, providing evidence against the claim that the null results simply originate from power problems.

**Fig. 1:**
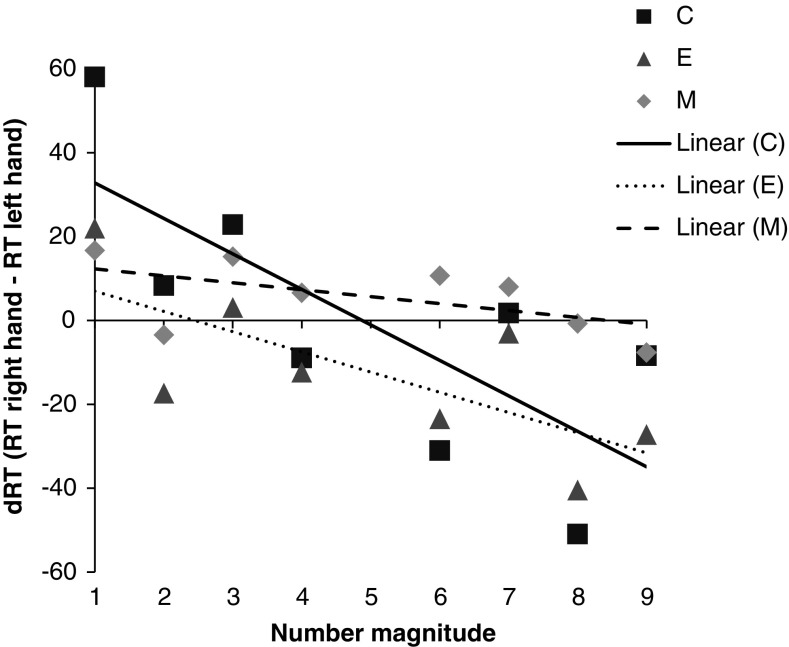
dRT and non-standardized SNARC slopes presented for each group separately. Slopes differ significantly from 0 only for the *C* (control) and *E* (engineers) groups but not for the *M* (mathematicians) group

Subsequently, we examined the presence of the SNARC effect in each group. Non-standardized slopes for each group are presented in Fig. 1. Crucially, there was no significant SNARC effect in the *M* group. Mean slope was −1.66 (SD = 5.93; ranging from −9.35 to 12.92) *t*_13_ = −1.05, *p* = .157 (one-sided). 9 out of 14 participants revealed a negative SNARC slope. In contrast, a significant SNARC effect was found in the *E* group (*t*_14_ = −3.12; *p* = .004; one-sided). Mean slope was −4.82 (SD = 6.0; ranging from −17.07 to 0.57). 13 out of 15 participants revealed negative slopes. Similarly, there was a significant SNARC effect in the *C* group (*t*_14_ = −4.01, *p* < .001; one-sided). Mean slope was −8.46 (SD = 8.17; ranging from −25.57 to 4.27). 13 out of 15 participants revealed negative slopes.

Subsequently, we tested for variance homogeneity across groups with the Bartlett test. The analysis revealed that there was no significant difference in variance between groups (*K*^2^ = 1.86; *df* = 2; *p* = .394). Group differences and null SNARC effects can thus not be attributed to more heterogeneity in the mathematicians group. Nevertheless, the exact tests for 2 × 2 tables did not reveal significant between-group differences in proportions of participants revealing negative slopes (*p*’s > .230).

### Correlates of the SNARC effect

In the next step, we aimed at testing whether the size of the SNARC effect correlated with other measures used in our study. All correlations are presented in Table 2.

**Table 2:** Correlation between measures of the SNARC effect (non-standardized slopes, standardized slopes, multiple regression results), participants’ RT characteristics, and Advanced Raven matrices total score

Measure	1	2	3	4	5	6	7	8
Non-standardized SNARC slope	–							
Mean RT	−0.26							
SD (RT)	−0.40**	0.82**						
Standardized SNARC Slope	0.79**	0.07	−0.02					
Multiple regression *R* ^2^	−0.24	−0.09	−0.06	−0.12				
Multiple regression—residual	−0.33*	0.64**	0.77**	−0.06	−0.33**			
Multiple regression—magnitude (SNARC)	1.0**	−0.26	−0.40**	0.79**	−0.24	−0.33*		
Multiple regression—parity (MARC)	0.18	0.10	−0.01	0.07	−0.09	−0.10	0.18	
Raven	0.25	−0.36*	−0.49**	0.11	−0.04	−0.44**	0.25	0.18

* *p* < .05, ** *p* < .01 (two-sided)

Although numerically larger than zero, the correlation of the individual SNARC effect slope with mean RT failed to reach significance. However, as in Cipora and Nuerk (2013), the SNARC effect correlated with intraindividual variability in RT. It is noteworthy that there was no correlation between the SNARC effect and the Advanced Raven Matrices score. Finally, for a full overview, correlations for each single group are separately presented in the “Appendix”.

### Further investigation of between-group differences in SNARC

Having explored the correlations between the SNARC effect and several other measures, we aimed at checking whether between-group differences in SNARC hold, when controlling for possible covariates. There was a significant bivariate correlation between SNARC slopes and intradindvidual variability in response times. Therefore, we conducted analysis of covariance (ANCOVA) to investigate, whether between-group differences in SNARC slopes still hold, when we control for this variable (see Tabachnick & Fidell, 2007; pp. 196–203 for rationale and recommendations regarding use of ANCOVA).

The main effect of the group remained when we controlled for intraindividual variability in RT (*F*_2, 40_ = 3.51; MSE = 39.71; *p* = .040, partial *η*^2^ = 0.15). The effect of the covariate intraindividual variability in RT on the dependent variable SNARC slope was also significant (*F*_1,40_ = 7.69; *p* = .008; partial *η*^2^ = 0.16). Pairwise between-group comparisons with Bonferroni-Holm correction revealed a significant difference between the *M* and the *C* group (*p* = .036), when controlling for intraindividual variability in RT.

We also conducted an ANCOVA controlling for the potential impact of fluid intelligence on the dependent variable. In that analysis, the main effect of group failed to reach significance (*F*_2,39_ = 2.21; MSE = 47.93; *p* = .124, partial *η*^2^ = 0.102). The effect of fluid intelligence was not significant as well (*F*_1,39_ = 0.22; *p* = .639, partial *η*^2^ = 0.006). This result must be treated with great caution, because it is very likely that in our quasi-experimental design the differences in fluid intelligence are associated with group assignment (see Tabachnick & Fidell, 2007; p. 200). We want to stress that this ANCOVA result is not conclusive and should normally not be conducted because assumptions for a meaningful interpretation of the covariate effect are violated: (1) there is no correlation between SNARC slopes and fluid intelligence at the whole sample level; and (2) there is no such correlation in any group. We will elaborate on this point in the “Discussion” section.

### SNARC and MARC effects: calculation using multiple regression approach

To examine a potential MARC effect (i.e., right-hand advantage for even numbers and left-hand advantage for odd numbers) together with the SNARC effect, individual multiple regressions were run on dRT (see Nuerk et al., 2005a, b, for the rationale). Odd numbers were coded as 0 and even numbers as 1. Non-standardized slopes were used for further analyses. In case of the magnitude predictor, the mean slope was −5.05 (SD = 7.20) and differed significantly from 0 (*t*_44_ = −4.66, *p* < .001; one-sided). Henceforth, even when controlling for parity, a significant SNARC effect was observed. The parity slope (mean = −22.43, SD = 66.97) also differed significantly from 0 (*t*_43_ = −2.22, *p* = .016; one-sided) revealing a significant MARC effect. Subsequently, SNARC and MARC slopes were examined (tested against 0) within each group. The results are summarized in Table 3. The results regarding the SNARC effect are very similar to those obtained for the one-predictor regression (described above). There was a significant SNARC effect in the *E* and the *C* group but there was no SNARC in the *M* group.

**Table 3:** SNARC and MARC effect estimates based on a multiple regression analysis across all three groups taken together

Group	Non-standardized SNARC slope			Non-standardized MARC effect		
	Mean	SD	Test against 0	Mean	SD	Test against 0
*M*	−1.66	5.92	*t*_13_ = −1.05, *p* = .157	−4.80	57.97	*t*_13_ = −0.31, *p* = .381
*E*	−4.82	5.99	*t*_14_ = −3.12, *p* = .004	−22.11	57.13	*t*_14_ = −1.50, *p* = .078
*C*	−8.46	8.17	*t*_14_ = −4.01, *p* < .001	−39.21	82.34	*t*_14_ = −1.84, *p* = .043

Slopes were tested against 0 with one-sample *t* tests (one-sided, for negative values), results of which are also presented in the table

Although the overall MARC effect over all groups was significant, the MARC effect reached significance only in the *C* group, probably due to power problems. In the next step, we compared groups with respect to the size of the SNARC and MARC effects. In case of the SNARC effect, there was a significant between-group difference, which is almost identical to the results of the ANOVA on slopes from the simple regression. In case of the MARC effect, there was no significant between-group difference (*F*_2,41_ = 0.95, MSE = 4495.06, *p* = .394, partial *η*^2^ = 0.04).

### Standardized SNARC slopes

In the last step of the analysis, we examined standardized SNARC slopes (i.e., within-participants Pearson correlation between number magnitude and dRT). Standardized slopes were Fisher-Z transformed prior to the analysis, so that they followed better a normal distribution.

At the group level, we found a significant SNARC effect (*t*_43_ = −4.32, *p* < .001). The mean was −0.36 (SD = 0.55). Analysis in groups taken separately revealed an analogous pattern of results as for the analyses on non-standardized slopes. There was no SNARC in the *M* group (*t*_13_ = −0.80, *p* = .438) with a mean slope of −0.15 (SD = 0.68). Contrarily there was a significant SNARC effect in the *E* group (*t*_14_ = −3.14, *p* = .008, mean slope −0.37, SD = 0.46) and the *C* group (*t*_14_ = −4.70, *p* < .001, mean slope −0.54, SD = 0.45). There were no significant between-group differences in standardized SNARC slopes (*F*_2,41_ = 1.97, MSE = 0.29, *p* = .153, partial *η*^2^ = 0.09).

## Discussion

In the presented paper, we aimed at investigating the relation between the SNARC effect and mathematical proficiency including a group of professional mathematicians. We recruited three groups of participants, professional mathematicians; professionals who use arithmetic in their everyday work, but who do not conduct research using mathematical reasoning itself; and controls, who are not or hardly ever required to use math in their everyday work.

Most importantly, in contrast to most previous studies, we found a significant between-group difference with respect to the SNARC effect, which was mainly driven by the professional mathematicians, whose SNARC effects have—to the best of our knowledge—never been studied before. Professional mathematicians did not reveal a significant SNARC effect, while the other two groups did. Professional mathematicians significantly differed from the other two control groups, while those two control groups with more or less arithmetic expertise did not differ from each other; this replicates earlier results of most studies before. This difference between mathematicians and control groups still held when various covariates were controlled for, such as RT characteristics (mean RT as well as intraindividual variability in RT). Within groups, fluid intelligence did not correlate with the SNARC, so that between-group differences in fluid intelligence cannot explain the SNARC effect, because if fluid intelligence determines the SNARC effect, it should do so within groups as well. The correlation at the sample level between fluid intelligence and SNARC was also not significant. The ANCOVA results controlling for fluid intelligence brought inconsistent findings, nevertheless they must be interpreted with great caution because ANCOVA assumptions were strongly violated (e.g., the covariate was not independent from the factor underlying group assignment) and the sample size was relatively small. In similar cases, several authors refrained from ANCOVA usage, when there is no zero-order correlation between a potential covariate and the dependent variable (e.g. Göbel, Moeller, Pixner, Kaufmann, & Nuerk, 2013).

The results also did not change substantially, when alternative methods of computing SNARC effects were used. We found the same pattern of results when magnitude slopes from multiple regression (i.e., controlling for the MARC effect) were analyzed. When we analyzed standardized SNARC slopes, the general pattern of results was similar: namely when compared to 0, professional mathematicians did not reveal a significant SNARC effect (contrary to the *E* and *C* groups). A notable difference between the analyses using non-standardized vs standardized slopes is that in the latter the between-group difference failed to reach significance. Standardized SNARC slopes consider the intraindividual variability within a subject, especially, how much dRT points are dispersed around the regression slope. So in principle the non-standardized regression slope could be almost 0; however, when all data points would be located almost exactly on the regression slope, the standardized slope would be very high. In essence, it is an index for how good the prediction of space-number associations by number magnitude is. However, it does not tell us much about how pronounced this association is. This is coded by the non-standardized slope, which reveals how many milliseconds faster a congruent spatial response is. In our data, the most likely explanation for the slight differences are high intraindividual variances in some participants. If those participants have high non-standardized SNARC slopes (e.g., in the *C* group), their non-standardized slopes might differ considerably from other groups but their predictions indexed by the standardized slopes might only be slightly different because they are corrected for their higher intraindividual variability.

Apart from group differences, the SNARC effect was related to response time characteristics (mean RT and intraindividual variability in RT). Nevertheless, this correlation was present only when non-standardized SNARC slopes were analyzed. The relation between SNARC and RT characteristics may therefore just be an artifact originating from a difference measure (i.e., dRT being the result of subtracting two RT) used to calculate SNARC (see Tzelgov, Zohar-Shai, & Nuerk, 2013, for a methodological critique of using non-standardized SNARC slopes). In the model proposed by Gevers et al. (2006), the relationship between mean RT and SNARC is explained in terms of the cognitive mechanisms underlying the SNARC effect. As we have shown (and as already pointed out in Pinhas et al., 2012; Tzelgov et al., 2013), this relationship may largely originate from the way slopes are calculated, not from the nature of the SNARC effect itself. Therefore, it seems that the relationship between SNARC and mean RT (as well as variability in RT) may not be a consequence of the cognitive processes underlying the SNARC effect (e.g., Gevers et al., 2006), but depend on the measure employed. As outlined above, fluid intelligence (Raven Matrices) scores differed between the *M* group and the other two control groups. Nevertheless, fluid intelligence did not correlate with SNARC slopes nor standardized SNARC slopes; so individual and group differences in SNARC are not driven by fluid intelligence. However, fluid intelligence correlated moderately with RT characteristics. This observation is in line with the results showing a correlation between intelligence and chronometric tasks in general (Deary, Der, & Ford, 2001).

For the linguistic MARC effect, the different groups did not differ from one another. The MARC effect did not correlate with any other measure. Thus, diverging effects for professional mathematicians were specific to the SNARC effect per se and could not be generalized to another effect in the study.

In sum, professional mathematicians differed in the SNARC effect from the control groups in virtually all analyses. This group difference could not be explained by different RT characteristics or fluid intelligence. It was specific to the SNARC effect, but did not generalize to the MARC effect. Between the two non-professional mathematicians groups, no significant differences in the SNARC effect were observed, despite strong differences in daily arithmetic experience. This replicates earlier results (e.g., Cipora & Nuerk, 2013). SNARC effects do not seem to vary (much) with arithmetic proficiency in the normal range. Only when relatively large (*n* > 35), gender-balanced samples are examined, one may expect to have significant statistical test results for relatively small effects (Hoffmann et al., 2014b). The probability of finding a relationship between math proficiency and the SNARC effect increases when extreme groups are recruited (professional mathematicians vs people with math difficulties, as in Hoffmann et al., 2014b).

Reasons for the small or non-significant SNARC variations with arithmetic proficiency have been discussed in detail elsewhere (e.g., Cipora & Nuerk, 2013, see also Patro et al., 2014; for different spatial-numerical associations, which may be differently related to arithmetic proficiency in children). Therefore, we only focus on why the SNARC effect in professional mathematicians is significantly weaker than in other groups and not significantly different from zero. Note, however, that in our study 9 out of 14 (64 %) mathematicians revealed negative SNARC slopes. The slopes were not significantly different from zero. Given the relatively small sample size, it is still possible that this non-significant result is due to power problems.

### Reasons for lack of/significantly reduced SNARC in professional mathematicians

There may be several reasons for a null or significantly reduced SNARC effect in mathematicians. Here we focus on possible differences in (1) domain-general cognitive abilities, (2) the nature of number representations, and (3) the embodied cognition perspective.

*Inhibition and/or cognitive control capabilities* Tasks measuring the SNARC effect are at some point influenced by inhibition processes. In incongruent trials (a smaller magnitude number has to be responded to with the right hand and a bigger magnitude number with the left hand), the natural spatial mapping (according to some views because of the number location on the Mental Number Line) has to be overcome by task instructions (Gevers et al., 2006). Recent data show that the efficiency of inhibition correlates with the SNARC effect (Hoffmann et al., 2014a). It is possible that mathematicians (already characterized by higher fluid intelligence) may also have better inhibition and cognitive control capacities, because not jumping to (i.e., inhibiting) premature conclusions without proof is what their daily work is about (see Embretson, 1995; for recent evidence on the relationship between cognitive control, working memory, and fluid intelligence see Chuderski, Taraday, Nęcka & Smoleń, 2012). According to this line of explanation, a directional spatial-numerical mapping as indexed by the SNARC effect may just be masked by effective cognitive control of interference, but not be absent in mathematicians per se.

Such a cognitive control account is supported by various related findings. First, cognitive control plays a major role in other number processing effects (Macizo & Herrera, 2011, 2013; Huber, Moeller, Nuerk, & Willmes, 2013; Huber, Klein, Willmes, Nuerk, & Moeller, 2014a; Huber, Mann, Nuerk, & Moeller, 2014c; Huber, Moeller, & Nuerk, 2014d; see also Nuerk, Moeller, Klein, Willmes, & Fischer, 2011; Nuerk, Moeller, & Willmes, 2015, for overviews). It would not be surprising if this extends to other numerical effects such as the SNARC effect as well. Second, selective attention has been shown to be a prerequisite for the SNARC effect (Nuerk et al., 2005b): Even though the magnitude of distractors was processed in an Eriksen task, there was no SNARC effect for those distractors, only for the targets being attended. Third, inhibition is related to other numerical effects (Gilmore et al., 2013) and the SNARC effect as well (Hoffmann et al., 2014a).

*More abstract processing in professional mathematicians* This account does not refer to domain-general characteristics as above, but is rather related to the more domain-specific characteristics of mathematicians. In other words, their numerical representations might differ from those in non-mathematicians in that they may just be more abstract.

It has been argued that the SNARC effect is strongly influenced by cultural and embodied experience, such as reading direction (Shaki et al., 2009), finger counting (Fischer, 2008; for a thorough discussion on factors influencing spatial-numerical associations see also: Fischer & Brugger, 2011; Göbel, Shaki, & Fischer, 2011). Possibly mathematicians—because of their daily routine with highly abstract concepts—have just overcome the cultural and embodied experiences which drive our default spatial directionality of magnitude. This could be tested in the future by examining other instances and paradigms of spatial-numerical association: Professional mathematicians with neglect may neglect smaller numbers, commonly on the left side of the number line, to a lesser extent.

*More flexible spatial*-*numerical representations* Another related, albeit slightly different, account is that rather than having no spatial association with numbers (because they are abstract), mathematicians possess much more flexible representations. In the literature, it was usually claimed that the spatial code is automatically activated when numbers are perceived (Fias, Lauwereyns, & Lammertyn, 2001). Nevertheless, it was demonstrated that under particular conditions number magnitude (in case of distracter numbers) can be processed semantically, but the spatial code is not activated (Nuerk et al., 2005a, see above). So, mathematicians may have strong spatial-numerical associations, however, they may map numbers to space in a highly flexible way. Therefore, default left-to-right mappings like in the SNARC effect may become weaker or disappear. This may be particularly the case in the parity judgment task, where relating numerical magnitude to space is by no means mandatory to accomplish the parity decision. Mathematicians possessing more flexible representations may simply not activate the spatial aspects that are irrelevant for the task demands. It is possible, however, that in a magnitude comparison task, when spatial coding of magnitude may be helpful, mathematicians also activate more spatial-numerical associations. Here we can only conclude that mathematicians do not activate them automatically, when magnitude is task irrelevant. Evidence for such an account comes from a recent unpublished study by Cohen Kadosh and colleagues.^4^ They observed that mathematicians are better in a number line estimation task (cf. Siegler & Opfer, 2003) for positive numbers. So, mathematicians may well be able to map numbers to space, but they might do so less automatically in a default direction.

*Stable but non*-*linear/non*-*horizontal numerical representations* It is also possible that a considerable proportion of mathematicians possess relatively stable but non-linear monotone or even non-monotone or non-horizontal spatial-numerical representations (bent lines, circular or irregular forms, vertical or radial associations). These representations may resemble those reported for persons with number-form synesthesia. Synesthesia may influence elementary numerical processing (Cohen Kadosh & Henik, 2007), including the SNARC effect (Sagiv, Simner, Collins, Butterworth, & Ward, 2006). This may also be the case in mathematicians.

*Embodied cognition explanation:*^5^ It is also possible that high math competence or arithmetic skills (characteristic of both professionals using math in their everyday work—the *E* group and mathematicians) leads to a reduced SNARC when compared to controls. If this is correct, *a smaller *SNARC effect should be found in the engineer group compared to the control group in the present study. This is in line with results described by Hoffmann et al. (2014a), where a group consisting of mathematics and engineering students revealed a significantly smaller SNARC effect than controls.

However, an opposite effect can be expected from an embodied cognition perspective. Possibly, the professional work requirements of engineers relate more strongly to spatial properties of the environments as well as a higher propensity of motion in space. Such kinds of activity may even enhance the spatial mapping of numerical representations. If this is correct, *a stronger *SNARC effect should be found in the engineer group compared to the control group in the present study.

If both mechanisms are operative for engineers and influence the SNARC effect in opposite directions, they may cancel each other out. This may lead to a null difference between engineers and controls. If the mechanisms do not fully cancel each other out, some differences between engineers and controls may be observed.

Nevertheless, mathematicians differ from controls. The reason may be that they have less embodied experiences of space-number associations, because their daily work relates to abstract concepts. Therefore, embodiment does not lead to enhanced space-number associations in the *M* group. As a consequence, only their higher math skills may influence the SNARC effect and may lead to a reduced effect, as compared to controls.

### Limitations of the presented study^6^

In the present study, we did not include objective measures of calculation skills or math expertise. Therefore, we cannot be sure whether the *M* group did not differ from the *E* group with respect to calculation skills. The pattern of possible differences in arithmetic performance between the *E* and the *M* group may be qualitatively different: engineers may practice calculation skills more, whereas mathematicians mostly focus on mental manipulations of abstract material. Several cases of double dissociations between mental calculation efficiency and math expertise have also been reported (for an overview, see Pesenti, 2005). So, while this study established that there exists a difference between professional engineers and professional mathematicians, it does not yet allow strong conclusions about the underlying nature of this difference.

Administration of tasks aimed at measuring flexibility and abstractness of representations would help answering such questions regarding the nature of representations in professional mathematicians. It would also be interesting to include measures of cognitive inhibition in order to directly test whether the *M* group outperforms other groups in this respect and for measures of complex problem solving skills (see Sonnleitner et al., 2013). These latter abilities seem to be particularly important for professional mathematicians and may also moderate spatial-numerical associations.

One must also keep in mind that the sample was not gender matched, precluding to test for an impact of gender on the SNARC effect. Males were reported to reveal a stronger SNARC effect (Bull et al., 2013). However, since the proportion of male and female participants did not differ between groups, between-group SNARC differences cannot be attributed to gender differences. Nevertheless, with the current design, it was impossible to trace the interaction effects of gender and math skills on spatial-numerical associations. Testing this research question would also require larger sample sizes, since gender differences tend to be rather small. All these issues need to be addressed in future studies.

Possible differences between the *E* and the *C* group also deserve further investigation. Because the Bayesian analyses revealed only weak evidence in favor of a null effect, there is some probability that a between-group difference may still exist, especially since it was shown by Bull et al. (2013) that between-group differences in the SNARC effect are relatively hard to detect (Cipora & Wood, 2012 for simulation data).

## Conclusions

The SNARC effect disappears or is significantly reduced in professional mathematicians and differs from control groups, even when these controls do a lot of arithmetic in their daily professional lives, such as engineers. A number of domain-general accounts, like higher cognitive control and inhibition, or domain-specific accounts, like more abstract number processing or more flexible spatial associations with number, may be responsible for these results. We suggest that further exploring the reasons of why extreme groups like mathematicians do not show a SNARC effect and differ from normal controls can give us more insight about the mechanisms responsible for the SNARC effect per se. More generally, a better understanding of how high domain expertise and long training in a particular domain influences cognitive processes may also be informative for other cognitive domains.

## Appendix: Correlations between measures presented for each group separately

**Table:**

Measure	Group	1	2	3	4	5	6	7	8
Non-standardized SNARC slope	*M*	–							
	*E*	–							
	*C*	–							
Mean RT	*M*	−0.03							
	*E*	−0.22							
	*C*	−0.43							
SD (RT)	*M*	−0.10	0.92**						
	*E*	−0.48	0.74**						
	*C*	−0.66**	0.81**						
Standardized slope	*M*	0.93**	0.20	0.17					
	*E*	0.95**	−0.12	−0.27					
	*C*	0.57*	0.14	−0.02					
Multiple regression *R* ^2^	*M*	0.13	−0.44	−0.45	0.02				
	*E*	−0.45	0.17	0.17	−0.38				
	*C*	−0.22	0.23	0.27	0.38				
Multiple regression residual	*M*	−0.35	0.77**	0.88**	−0.04	−0.49			
	*E*	−0.08	0.48	0.59*	−0.01	−0.35			
	*C*	−0.80**	0.69**	0.82**	−0.20	0.19			
Multiple regression—magnitude (SNARC)	*M*	1.0**	−0.03	−0.10	0.93**	0.13	−0.35		
	*E*	1.0**	−0.22	−0.48	0.95**	−0.45	−0.08		
	*C*	1.0**	−0.43	−0.66**	0.57*	−0.22	−0.80**		
Multiple regression—parity (MARC)	*M*	0.47	0.01	−0.18	0.27	0.28	−0.32	0.47	
	*E*	−0.07	0.43	0.45	−0.03	−0.25	0.24	−0.07	
	*C*	0.02	0.05	−0.12	−0.23	−0.20	−0.14	0.02	
Raven	*M*	0.24	−0.59*	−0.64*	0.01	0.38	−0.72**	0.24	0.19
	*E*	0.09	−0.67**	−0.56*	−0.05	−0.38	−0.41	0.09	−0.20
	*C*	−0.06	0.21	−0.01	−0.13	0.23	−0.21	−0.06	0.24

* *p* < .05 (two-sided), ** *p* < .01 (two-sided)

## Footnotes

1 There are numerous anecdotal reports showing that outstanding mathematicians (e.g., Stanisław Mazur, one of the most famous Polish mathematicians) had difficulties with simple arithmetic in everyday life.

2 We did not include physicists to any group because in many cases their work mostly comprises mathematics but on the other hand is not mathematics per se.

3 Note that results are virtually the same in all instances if two-sided tests are applied except from MARC effect in the C group.

4 We are grateful to Roi Cohen Kadosh, who shared information about this yet unpublished study with us.

5 We would like to thank Martin Fischer for this suggestion.

6 We would like to thank the editor and two anonymous reviewers who suggested several points to be discussed in this section.
